# Hydrophobic Cellulose Acetate Aerogels for Thermal Insulation

**DOI:** 10.3390/gels8100671

**Published:** 2022-10-19

**Authors:** Sizhao Zhang, Zhouyuan Yang, Xing Huang, Jing Wang, Yunyun Xiao, Junpeng He, Jian Feng, Shixian Xiong, Zhengquan Li

**Affiliations:** 1 Polymer Aerogels Research Center, Jiangxi University of Science and Technology, Nanchang 330013, China; 2 Postdoctoral Research Station on Mechanics, College of Aerospace Science and Engineering, National University of Defense Technology, Changsha 410073, China; 3 Science and Technology on Advanced Ceramic Fibers and Composites Laboratory, National University of Defense Technology, Changsha 410073, China

**Keywords:** cellulose acetate aerogel, hydrophobic treatment, water contact angle, perfluorodecyltriethoxysilane

## Abstract

As naturally derived material, cellulose aerogels have excellent thermal insulation properties due to their unique high porosity and three-dimensional mesoporous structure. However, its hydrophilic properties limit its application in the field of building insulation. Here, we propose a method to prepare high hydrophobicity by adopting the sol-gel method and chemical vapor reaction strategy using cellulose acetate type II as raw material and 2,4-toluene diisocyanate as the cross-linking agent. Thermal properties of cellulose acetate aerogels (CAAs) were measured, where pyridine was the catalyst, acetone was the solvent, and perfluorodecyltriethoxysilane (PFDS), hexamethyldisilazane (HMDS), and methyltriethoxysilane (MTES) were used as hydrophobic agents (by process hydrophobic test). Compared with MTES-modified cellulose acetate aerogels (M-CAAs) and HMDS (H-CAAs)-modified cellulose acetate aerogels, PFDS-modified (P-CAAs) cellulose acetate aerogels are the most hydrophobic. By implementing hydrophobic modification of PFDS both inside and outside the structure of cellulose acetate aerogels, the water contact angle can reach up to 136°, strongly demonstrating the potential of PFDS as a hydrophobic agent. The results show that the thermal conductivity and compressive strength of cellulose acetate aerogel with the best hydrophobic properties are 0.035 W m^−1^ K^−1^ at normal pressure and 0.39 MPa at 3% strain, respectively. This work shows that the highly hydrophobic cellulose acetate aerogel has potential as a waterproof material in the field of building thermal-insulation materials.

## 1. Introduction

Cellulose aerogel is characterized by low density, high porosity, and high specific surface area [1,2]. Research has shown that density and porosity are the main factors affecting the thermal conductivity of aerogel materials, with their thermal insulation properties decreasing in accordance with an increase in porosity and a decrease in density [3,4]. Cellulose acetate, which served as the basic synthetic raw material in the present experiment, is abundant in nature and has various beneficial properties, including renewability, good biocompatibility, and excellent biodegradability, thereby overcoming health and environmental problems associated with traditional building insulation materials. Gong et al. [5] reported cellulose aerogels with the following specifications: density of 0.009–0.137 g/cm^3^, specific surface area of 120–230 m^2^/g, and thermal conductivity of 0.04–0.075 W m^−1^ K^−1^. Yuan et al. [6] and Kobayashi et al. [7] reported a new type of aerogel with a novel structure, which combined good heat insulation, optical transparency, and mechanical toughness. This aerogel consists of three-dimensionally ordered nanofiber skeletons of liquid–crystalline nanocellulose [8,9], and the lowest thermal conductivity is 0.018 W m^−1^ K^−1^ at a density of 0.017 g/cm^3^. Cellulose aerogels have a strong potential to serve as construction insulation materials due to their low thermal conductivity [10], as typical thermal conductivity of conventional building insulation materials can reach up to 0.03 W m^−1^ K^−1^ [11,12,13]. Cellulose acetate is one of the first commercially available and fully soluble cellulose derivatives for preparation of cellulose acetate aerogels [14]. Cellulose acetate aerogels also have advantages over conventional insulation materials in terms of their biodegradability and renewability [15]. All the properties discussed above enable cellulose acetate aerogels to be a good candidate for building insulation materials.

Additionally, low thermal conductivity and compressive strength, building insulation materials must also maintain hydrophobicity to ensure that the material can be safely applied in wet conditions [16,17,18]. The hydrophilicity of hydroxyl groups that remain following material preparation causes cellulose acetate aerogels to easily absorb water in a humid environment [19,20,21]. Thus, the hydrophobic properties of cellulose acetate aerogels must be improved via surface modification using hydrophobic agents before they can be used in the building industry.

According to the theory of hydrophobic alteration, there are two major approaches to achieve hydrophobicity. One is to construct a nano/micron structure layer on the surface of the material to inhibit the spread of liquid water, and the other is to coat the surface of the material with a low surface energy material to reduce the capillary force at the liquid–solid interface. This suppresses the destruction of the microscopic pore structure of the material by capillary forces [22,23,24]. Based on these two approaches discussed, several methods for hydrophobic modification of cellulose acetate aerogel materials were proposed including the sol-gel method, solution immersion method, chemical etching, electrochemical deposition, and chemical gas inverse response [25]. However, the sol-gel method has a problem with nanoparticle dispersal, and the solution immersion method has a problem with uneven growth of the nanoparticles. Chemical etching and electrochemical deposition always rely on complicated processes, expensive equipment, and high costs [26,27]. In contrast, the chemical vapor-phase reaction method is feasible and straightforward. This method employs gas-phase diffusion. Thus, hydrophobic modification is uniform, and the resulting hydrophobicity of the material is good. Therefore, in our study, we prepared a highly hydrophobic cellulose acetate aerogel using the chemical vapor-phase reaction method.

Traditional hydrophobic agents consist mostly of long-chain alkanes or long-chain silanes, both of which have longer carbon chains and contain groups, such as methyl and ethyl groups, which confer good hydrophobic properties [28]. The hydrophobic properties of different groups are ranked from high to low [29], as follows: -CF_3_, -CF_2_-, -CH_3_, and -CH_2_-. Perfluorodecyltriethoxysilane (PFDS), a fluorine-containing, long carbon chain silane with a variety of hydrophobic groups, including -CF_3_, -CF_2_-, -CH_3_, and -CH_2_-, exhibits better hydrophobic modification ability.

The aim of the present study was to investigate the performance of PFDS as a hydrophobic agent. With this aim in mind, we investigated the hydrophobic effects of PFDS and the water contact angle in P-CAAs. We also investigated the impact of different hydrophobic agent/aerogel mass ratios (0.1–0.4) on weight gain rates and moisture adsorption. We describe a strategy for the preparation of highly hydrophobic cellulose acetate aerogels that can be widely used in the building insulation industry [30,31].

## 2. Results and Discussion

### 2.1. Microstructure Texture

**Figure 1:**
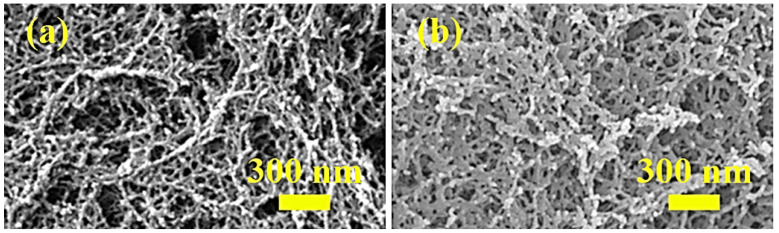
FESEM images of (**a**) CAAs and (**b**) P-CAAs.

The microstructure of CAAs before and after hydrophobic modification is shown in Figure 1. Before hydrophobic modification, the CAAs had a three-dimensional nanoporous network structure, with a pore size of 50–100 nm. After hydrophobic modification, the P-CAAs maintained a good nanoporous network structure, indicating that PFDS does not destroy the nanoporous network structure of CAAs. As shown in Figure 1, the network structure of the modified aerogels became denser, which may result from the hydrophobic agents that promote network cross-linking in the gel.

**Figure 2:**
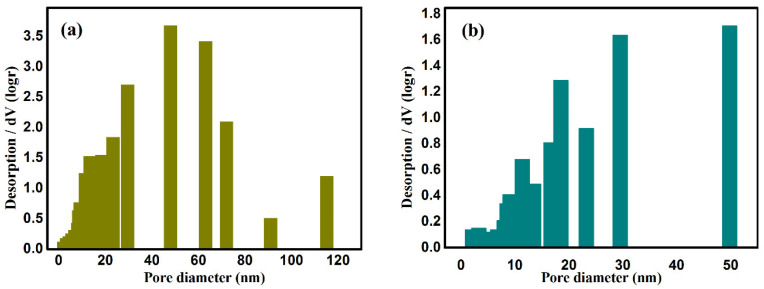
Pore size distributions, pore volume, and specific surface area before (**a**) and after (**b**) hydrophobic modification.

The pore size and distribution in the CAAs before and after modification by PFDS are shown in Figure 2. The pore size in CAAs prior to hydrophobic modification is mainly concentrated in the range 40–80 nm. The pore size in the P-CAAs was in the range 20–50 nm, in line with the SEM observations.

### 2.2. Cross-Linking Reaction and Hydrophobic Reaction

**Figure 3:**
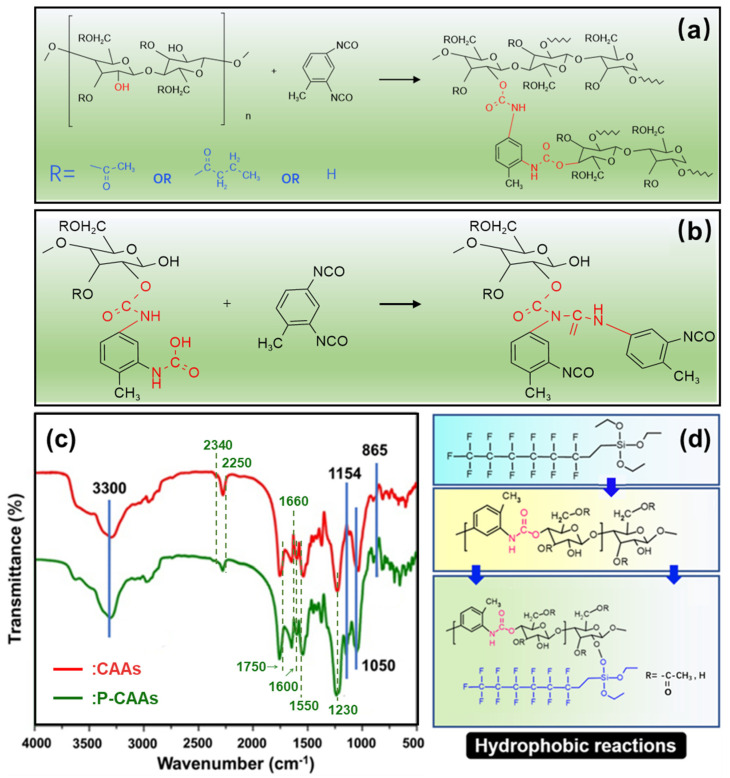
(**a**) First-form cross-linking reaction of cellulose acetate aerogel and (**b**) second-form cross-linking reaction of cellulose acetate aerogel. FTIR spectra of CAAs samples (**c**) (red) and P-CAAs (green) and (**d**) PFDS chemical modification reaction.

Under the catalysis of pyridine, 2,4-diisocyanated toluene ester can cross-link with CA to achieve gelation, as shown in Figure 3a. Under the catalysis of pyridine, the hydroxyl group (–OH) on the main molecular chain of CA reacts with the cyanate ester group (–N=C=O) on the cross-linking agent to form the urethane bond (–CONH). When the cross-linking agent is excessive, as shown in Figure 3b, the hydrogen transfer reaction also occurs between the cross-linking agent and the formed urethane group to form the urea formate, which promotes the cross-linking between raw materials. At the same time, the hydroxyl group on the cellulose acetate molecular chain and the formed urethane bond create a strong hydrogen bond between urea formate groups. The CA molecules and cross-linking agents then form cross-linking under joint action of the chemical cross-linking reaction and hydrogen bond, and finally form a polymerization network structure.

The CA molecule is derived from the esterification of cellulose, a natural polymer, which maintains the long chain structure of the macromolecule of the natural polymer itself. There are many crystalline regions and some amorphous regions inside, which are nanofibrous in acetone sol. The probability of mutual contact between cross-linking units is increased, and some areas are physically entangled at the same time under the action of hydrogen bonds. Finally, short-range cross-linking units are gradually polymerized to form a long-range three-dimensional network gel.

**Figure 4:**
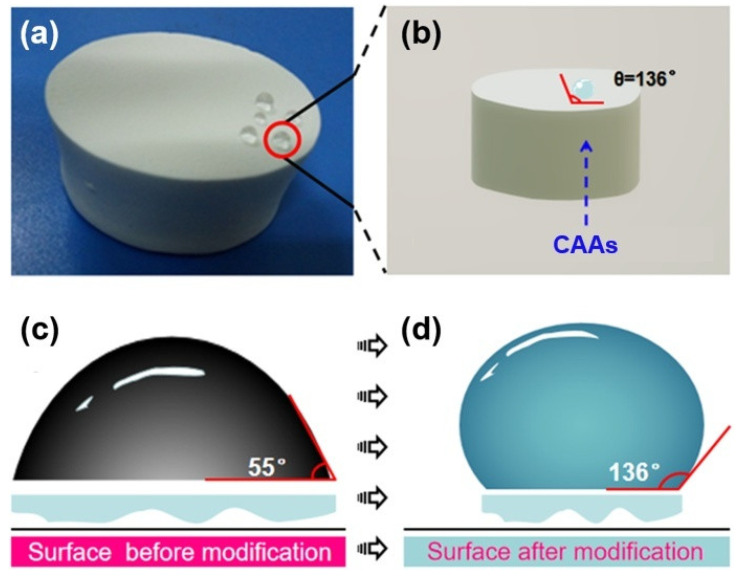
(**a**) A photograph of the hydrophobic CAAs and (**b**) a schematic of the hydrophobic surface. Schematic diagram of the water contact angle (**c**) before and (**d**) after hydrophobic modification.

At the same time, a water contact angle instrument was used to characterize the CAAs after hydrophobic treatment. Figure 4 shows the comparison of the contact angle of CAAs before and after hydrophobic modification. It can be seen from the figure that CAAs have obvious hydrophilicity before hydrophobicity, and the water contact angle is shown as 55°, and after the hydrophobic treatment, the water contact angle becomes 136°. The hydrophilicity of CAAs was obviously changed, making it somewhat hydrophobic. The fundamental reason behind this is that the treatment of the hydrophobic agent reduces the hydroxyl group, and at the same time, because the hydrophobic binding of the hydrophobic agent and CAAs is a chemical covalent bond, it has a strong binding ability, which makes CAAs have better hydrophobic stability.

The hydroxyl group in the molecular chain of the CAAs was dehydrogenated and reacted with PFDS to connect the fluorinated long carbon chain to the main chain of the CAAs. Figure 3c shows the FTIR spectra of the CAAs samples before and after the hydrophobic modification treatment. As shown in Figure 4c, after hydrophobic modification of the aerogel, two types of new absorption peaks appeared in the infrared absorption curve of the modified aerogels, and the peaks for 1154 and 865 cm^−1^ correspond to the C–F bond and –Si–O–C bond, respectively. These results indicated that the fluorine-containing long-chain silane was successfully connected to the main chains of the aerogel. At the same time, the absorption intensity at the 3300 and 1050 cm^−1^ peaks correspond to C–O bonds at –OH and –OH, respectively, indicating that the hydrophobic treatment reduced residual hydroxyl groups inside the CAAs. The cellulose acetate aerogel used in this study is a polymer that is cross-linked with urethane bonds. Some hydroxyl groups remain on the main chain. The hydrophobic reaction of the cellulose acetate aerogel with PFDS is shown in Figure 3d.

In order to verify whether the cross-linking reaction of the prepared cellulose acetate aerogel produces urethane bond and carbamate, the aerogel was characterized by FTIR. Figure 3c is the FTIR spectrum of CAAs compared to recent research [32]. There are seven characteristic absorption peaks in the figure: 3330 cm^−1^ represents the stretching vibration of N-H; 2340 cm^−1^ represents the stretching vibration of the cyanate ester group (–N=C=O); 1750 and 1660 cm^−1^ represent the stretching and bending vibration of the carbonyl group (–C=O) in the ester group; 1600 cm^−1^ represents the stretching vibration of the benzene ring; 1550 cm^−1^ represents the stretching vibration of C–N and the bending vibration of N-H; and 1230 cm^−1^ represents the stretching vibration of C–O. Among them, 1750 and 1230 cm^−1^ are common to cellulose acetate aerogels, indicating that ester groups exist in both of them, which is corresponding to their molecular structures. Compared with cellulose acetate, there are five more absorption peaks in the FTIR curve of cellulose acetate aerogel: 3330, 2250, 1600, 1550, and 1230 cm^−1^, indicating that toluene isocyanate successfully combines with cellulose acetate molecules, and there is an ammonia ester bond (–O–CONH) in the cellulose acetate aerogel. Correspondingly, the absorption intensity of the red line, 2340, 1660, 1600, and 1550 cm^−1^ correspond to the absorption peak shown in Figure 3c, which confirms that the urethane bond reacts with the excessive cross-linking agent to form urea formate. At the same time, this also proves the existence of noncovalent bonds in the cross-linking reaction of CAAs, which is also a hydrogen bond between the hydroxyl molecular chain on cellulose acetate and the polyurethane bond, thus forming a formate urea group.

### 2.3. Hydrophobicity Evaluation

To explore the optimal hydrophobic treatment conditions, the effects of two parameters, the hydrophobic agent/aerogel mass ratio and hydrophobic modification time, on the hydrophobic properties of the cellulose acetate aerogel were evaluated [33]. The hydrophobic agent (PFDS)/aerogel mass ratio is defined as the ratio of mass of the hydrophobic agent to that of the cellulose acetate aerogel. The ratios selected were 0.1, 0.2, 0.3, and 0.4. The hydrophobic time was set at 1, 2, 3, and 4 d. The hydrophobic modification temperature was 70 °C.

**Figure 5:**
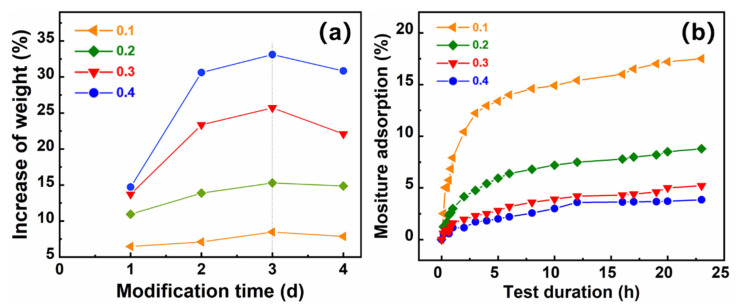
(**a**) Curves showing the influence of modification time on increase in weight at different hydrophobic agent/aerogel mass ratios, and (**b**) curves indicating the influence of moisture and test duration on different hydrophobic agent/aerogel mass ratios.

First, we analyzed the effect of the hydrophobic modification time (days) with the different hydrophobic agents at different hydrophobic agent/aerogel mass ratios on the degree of hydrophobicity. Figure 5a shows the curve of increasing weight-gain rate with modification time for different hydrophobic/aerogel mass ratios. In the initial stage of the hydrophobic reaction, the sample gained weight rapidly, and the hydrophobic agent penetrated rapidly into the aerogel network, where it bound rapidly to the hydroxyl groups in the cellulose acetate aerogel molecular chain. After 3 d of treatment with the hydrophobic agent, saturation was reached, and the sample was essentially stable. The curves for the relationship of the increase in weight and modification time were presented to indicate the complete hydrophobic reactions by mass equilibrium. Thus, 3 d of treatment with the hydrophobic agent was sufficient for the hydroxyl groups inside the cellulose acetate aerogel to be modified.

After the hydrophobic treatment, moisture tests were conducted at fixed temperature (50 °C) and humidity (95%) to confirm the success and completion of the hydrophobic treatment. The hydrophobic CAAs retained a certain amount of moisture by adsorption, as can be seen in Figure 5. According to analyses of hygroscopicity of porous materials, the moisture adsorption observed herein can be attributed to physical adsorption of liquid water in the pores of the aerogel and chemical bonding (hydrogen bonding) of a small amount of liquid water with hydroxyl groups. Furthermore, the smaller the mass ratio of a hydrophobic agent/aerogel, the more the hygroscopicity of the hydrophobic cellulose acetate aerogel in the initial phase, indicating that the more residual hydroxyl groups there are, the larger the internal pore volume. As the hydrophobicity test time was extended, the mass moisture adsorption gradually stabilized. The greater the hydrophobic agent/aerogel mass ratio of the hydrophobic CAAs, the smaller the saturated mass moisture adsorption rate, which corresponded to fewer hydroxyl groups and a smaller pore volume. There was no apparent difference in the hydrophobic performance at mass ratios between 0.3 and 0.4. Therefore, a hydrophobic agent/aerogel mass ratio of 0.3 is optimum to reduce the cost.

**Figure 6:**
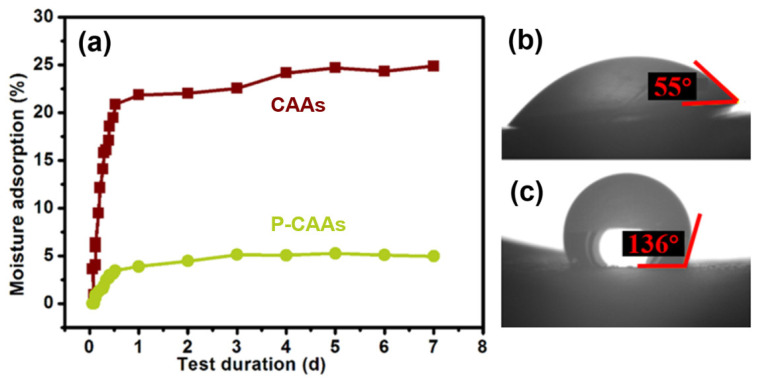
(**a**) Curves for the moisture absorption of the CAAs and P-CAAs as a function of the treatment duration. (**b**) Water contact angle of CAAs. (**c**) Water contact angle of P-CAAs.

According to the optimal hydrophobic reaction conditions (i.e., hydrophobic agent/aerogel mass ratio of 0.3 and hydrophobic treatment for 3 d), a hydrophobic cellulose acetate aerogel was prepared. A water contact angle tester was used to characterize the hydrophobic aerogel and obtain the water contact angles measurements of the CAAs before (Figure 6b) and after (Figure 6c) hydrophobic modification. Before the hydrophobic treatment with PFDS, the cellulose acetate aerogel was obviously hydrophilic, with a water contact angle of 55°. After treatment with the hydrophobic agent, the water contact angle increased to 136°, which significantly changed the cellulose acetate aerogel from the hydrophilic state to the hydrophobic one. This hydrophobicity is comparable to those recently reported in the literature [34,35]. The hydrophobic treatment reduced the hydroxyl groups and pore size, resulting in slower growth of the mass hygroscopicity of the aerogel and a lower saturation mass hygroscopicity.

Furthermore, we tested the hydrophobic stability of the treated aerogel. The water interaction angle and saturated mass moisture absorption of the hydrophobic cellulose acetate aerogel post-treatment with the hydrophobic agent were compared. In all the tests, the treatment duration was 7 d, and the aerogel samples were dried after each hydrophobicity test. The results showed that after multiple hydrophobicity tests, the water contact angles and saturated mass moisture absorption rates of the hydrophobic cellulose acetate aerogel remained basically unchanged, pointing to good hydrophobic performance and aerogel stability. These findings are similar to those of Yang et al. [33]. The hydrophobic binding of the hydrophobic agent and cellulose acetate aerogel via a chemical covalent bond may explain the strong binding ability. As the modified chemical bonds in aerogels do not change when the gels are exposed to a temperature of 50 °C, surface-modified hydrophobic cellulose acetate aerogels at this temperature have improved stability and improved hydrophobic properties [36].

**Figure 7:**
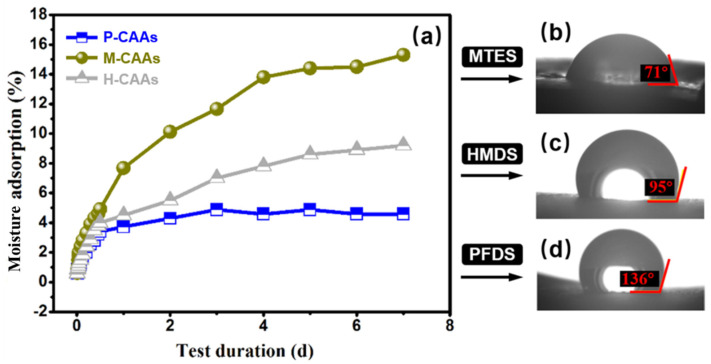
(**a**) Water contact angles and moisture absorption rates for different hydrophobic agents as a function of the hydrophobic test time. (**b**) Water contact angle of H-CAAs. (**c**) Water contact angle of H-CAAs. (**d**) Water contact angle of P-CAAs.

Compared to methyl-containing or ethyl-containing hydrophobic agents, such as HMDS and MTES, PFDS has better hydrophobic modification performance. Figure 7 shows the water contact angles and moisture absorption rates of the hydrophobic agents PFDS, MTES, and HMDS as a function of the hydrophobic test duration. Obviously, the hydrophobic property of cellulose acetate aerogels with PFDS modification treatment was the highest among the others (including those modified with MTES and HMDS, as presented in Figure 7), suggesting the feasibility for the adopted PFDS containing fluor in great measure. It shows that MTES had poor hydrophobic effect due to the cellulose acetate aerogel exhibiting a degree of hydrophilicity after treatment. The hydrophobic modification effect of HMDS was better than that of MTES, as the water contact angle increased from 55° to 95°. In the moisture test, after hydrophobic modification, the saturated mass moisture absorption of HMDS and MTES decreased with increasing time. However, the modification effects of PFDS showed the highest contact angle and lowest moisture adsorption. Fluorine-containing hydrophobic agents are more reactive with hydroxyl groups in cellulose acetate aerogels. Thus, the hydrophobicity of CAAs treated with fluorine-containing hydrophobic agents (PFDS) is better than that of HMDS and MTES. The PFDS coating decreases the pore diameter of the material and consequently reduces the physical adsorption of liquids. As a result, the saturated mass hygroscopicity becomes relatively smaller.

### 2.4. Thermal Conductivity Test

**Figure 8:**
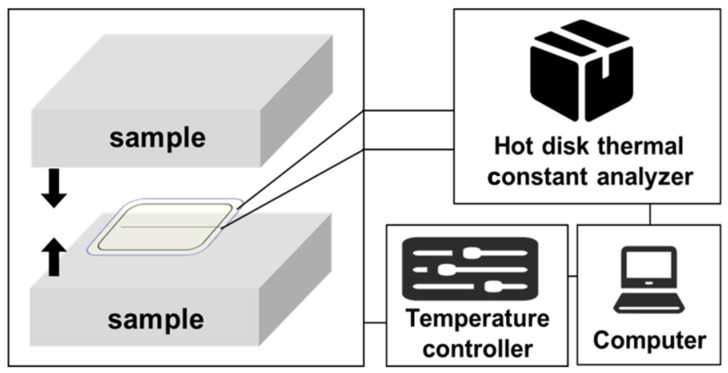
Schematic diagram of the experimental setup for thermal conductivity testing.

The thermal conductivity of cellulose acetate aerogel was tested by using a Sweden TPS 2500 Hot Disk thermal constant analyzer. The test temperature was 25 ℃. The thermal conductivity of the material was tested under atmospheric pressure. The plain aerogel samples were processed to a specific size (φ20 × 20 mm^3^), with two samples per group. The schematic diagram of the experimental setup is shown in Figure 8.

The test principle is to place the Hot Disk probe between two test materials, and by passing a constant power current into the test probe, the test material and the surface of the Hot Disk probe have a certain temperature rise. Conductivity is closely related. Since the temperature rise of the probe surface can be monitored by the resistance change in the nickel wire in the probe, the probe is both a heat source and a temperature sensor, so the thermal conductivity of the test material can be obtained by monitoring the temperature-rise curve of the probe surface. Finally, the thermal conductivity of the experimental sample was measured to be 0.035 W m^−1^ K^−1^.

### 2.5. Compressive Strength and Its Analysis

**Figure 9:**
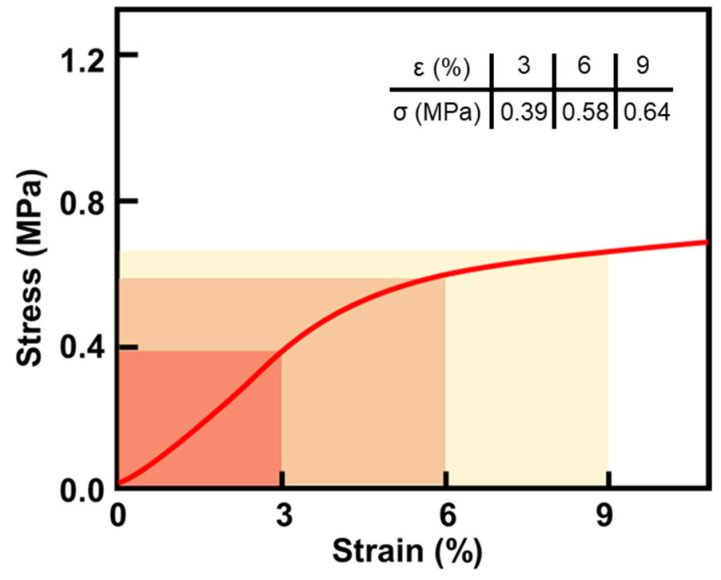
Compressive stress–strain curve of CAAs.

The compressive strength and modulus of cellulose acetate aerogels were tested by a WDW-100 electromechanical universal testing machine. For aerogel materials, the compressive strength is often expressed by the stress values corresponding to 10% and 25% strain. For testing, a preprocessed sample with a specific size (20 × 20 × 20 mm^3^) (five samples per group) was placed at the center of the pressure plate of the testing machine and pressurized vertically at a loading rate of 1 mm/min. The final compression data are shown in Figure 9. From the Figure 9, we can see that the stress of CAAs reached 0.39 MPa under 3% strain.

Combined with the microstructure analysis of cellulose acetate aerogel (Figure 1), CAAs have good ability to resist compressive stress, because the internal cross-linking reaction of the gel is relatively complete, the skeleton growth in the network becomes relatively thick, and the lamellae are relatively thick. As the structure expands and widens, the internal network of the gel gradually increases its density.

## 3. Conclusions

In conclusion, firstly, cellulose acetate aerogels were successfully prepared by the sol-gel method using cellulose acetate type II as raw material. Then, CAAs with high hydrophobicity was successfully prepared by chemical vapor reaction by using PFDS, MTES, and HMDS as hydrophobic agents. Among them, the hydrophobicity of P-CAAs is better than the other two. By hydrophobically modifying PFDS inside and outside the CAAs structure (still maintaining high porosity), the optimal water contact angle reached 136°, which strongly demonstrated the potential of PFDS as a hydrophobic agent. After treatment with PDFS, the contact angle of water increased to 136°. We speculate that in addition to the covalent cross-linking reaction, there is noncovalent cross-linking, which is one of the reasons for the high performance of hydrophobicity. It prevents liquid water droplets from gathering on the surface of the aerogel, and significantly changes the surface properties of CAAs, transforming it from a hydrophilic state to a hydrophobic state. Finally, the thermal conductivity and compressive strength of CAAs with the best hydrophobic properties were tested under normal conditions. We anticipate that these highly hydrophobic CAAs will be used for thermal insulation in energy efficient buildings.

## 4. Experimental Section

### 4.1. Materials

**Figure 10:**
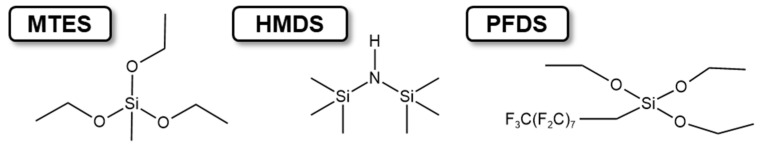
The chemical structure of PFDS, HMDS, and MTES.

Cellulose acetate (about 56% acetyl content) was purchased from Shanghai Jinshan Ting New Chemical Reagent Factory (Shanghai, China). Acetone (99.8%) and absolute ethanol (AR) were purchased from the Chemical Reagent Factory of Hunan Normal University (Changsha, China), and 2,4-tolylene diisocyanate (AR) was fabricated from Aike reagent (Chengdu, China). Pyridine (AR), perfluorodecyltriethoxysilane (PFDS), hexamethyldisilazane (HMDS), and methyl triethoxysilane (MTES), as shown in Figure 10, were purchased from Shanghai Sinopharm Chemical Reagent Co., Ltd. (Shanghai, China). All chemical reagents were used without further purification. In the absence of specific instructions, deionized water was used in all the experiments.

### 4.2. Synthesis of Cellulose Acetate Aerogel

Cellulose acetate, also known as cellulose acetate, is a common cellulose esterification derivative substance, which is a derivative product of cellulose esterification treatment.

According to the degree of esterification, cellulose acetate can be divided into cellulose diacetate and cellulose triacetate, of which cellulose triacetate is a complete esterification product; the hydroxyl groups on the cellulose molecular chain are esterified by acetate anhydride into acetate group, esterification degree of 280–300, which is easily soluble in dichloromethane, methyl acetate, and other solvents. The cellulose diacetate is a partial esterification product; the esterification degree is 220–270, and the hydroxyl group of some sites on the molecular chain can be retained, which has good organic solvent solubility, especially in acetone, and the solubility is particularly good.

In the preparation of the cellulose acetate aerogel, 2,4-dithionate was used as the catalyst for the cross-linking agent acrylamide, and acetone was used as a solvent. Cellulose acetate/acetone solution was used as the basic synthetic raw material. Cellulose acetate was added to acetone at room temperature and mixed well by stirring until the solution was clarified. Pyridine and 2,4-tolylene diisocyanate were then gradually added to the solution for 30 min, eventually resulting in a cellulose acetate/acetone sol. When bubbles began to emerge in the cellulose acetate/acetone mixture, stirring was stopped. The sol was then placed in a bespoke plastic mold (Φ50 × 30 mm^3^) and sealed with cling film. It was then left to stand at room temperature and pressure to obtain the gel.

The prepared cellulose acetate/acetone gel was then sealed and placed in a heated water bath for aging for 7 d, keeping the temperature at 45 °C. During the aging process, a small amount of acetone was added to the surface of the gel to prevent it from cracking. After the gel reached a certain strength, acetone was added as a solvent and replaced three times to remove unreacted raw materials inside the gel.

The aged cellulose acetate/acetone gel was placed in an autoclave for supercritical drying, with the gel submerged in acetone solvent to prevent cracking. Subsequently, a mixed fluid of carbon dioxide and the solvent was released every half hour. After all the solvent had been released, the pressure relief valve was opened to allow the pressure to return to normal. The gel was allowed to cool at room temperature before removing it from the autoclave, ultimately gaining the aerogels.

### 4.3. Hydrophobic Treatment

**Figure 11:**
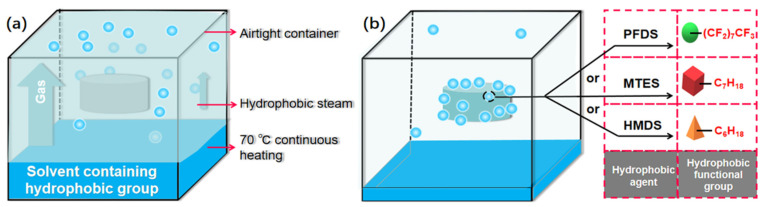
(**a**) Schematic diagram of the initial stage and (**b**) final stage of hydrophobic modification.

First, the prepared cellulose acetate was heated in an oven at 70 °C for 2 h. A wire mesh was then suspended in a closed container, and the cellulose acetate aerogel was placed on the suspended mesh. The hydrophobic agent was then placed at the bottom of the container, without any direct interaction between the cellulose acetate and the hydrophobic agent. The temperature in the closed container was set to 70 °C. The surface of the cellulose acetate aerogel was modified by evaporation of the hydrophobic agent in the container. The final hydrophobic cellulose acetate aerogel was collected after 3 d (Figure 11).

### 4.4. Characterizations

The Fourier-transform infrared (FTIR) spectra of all the dried specimens were observed at 400–4000 cm^−1^ using pressed KBr pellets on a spectrophotometer (Spectrum Frontier; PerkinElmer Co., Waltham, Massachusetts, USA). The surface texture and appearance of the prepared specimens were scanned using a field-emission scanning electron microscope (Helios Nanolab 600i, FEI Co., Shanghai, China) operated at 10 kV. The water contact angles of the hydrophobic cellulose acetate aerogel were determined using a contact angle tester (SL-200B; Shanghai Solon Information Technology Co., Ltd., Shanghai, China). A Programmable Constant Temperature and Humidity Tester (BE-TH-150D8; Dongguan Bell Co., Dongguan, China) was used to test mass moisture absorption. The test temperature was 50 °C, the air humidity was 95%, and the inside air remained free flowing. The specific surface area and pore structure of the cellulose acetate aerogel were determined using a physical adsorb-iQ2-MP (Autosorb-iQ2-MP; Conta Instruments, Inc. Shanghai, China). Thermal conductivity in normal pressure was tested by Hot Disk TPS 2500 (Sweden Hot Disk Co., Ltd. & KAITS Kegonas Instruments Trading Shanghai Co., Shanghai, China) apparatus with a 5465 sensor. The mechanical property was measured by using a universal testing machine (WDW-100, China; Shandong Wanchen Co., Shandong, China) with a load cell of 1 kN, and the compression rate was fixed at 0.5 mm/min.

## Data Availability

Data are available from the authors. Samples of the compounds are available from the authors.
